# Prevalence of human immunodeficiency virus infection in a cohort of tuberculosis patients at Metema Hospital, Northwest Ethiopia: a 3 years retrospective study

**DOI:** 10.1186/s13104-016-2004-8

**Published:** 2016-03-29

**Authors:** Daniel Tarekegne, Muhabaw Jemal, Tadesse Atanaw, Ashenafi Ebabu, Mengistu Endris, Feleke Moges, Belay Tessema, Tekalign Deressa

**Affiliations:** School of Biomedical and Laboratory Sciences, University of Gondar, Gondar, Ethiopia

**Keywords:** HIV, Tuberculosis, Co-infection, Trends, Metema, Northwest Ethiopia

## Abstract

**Background:**

Ethiopia is one of the countries that are highly affected by dual epidemics of human immunodeficiency virus (HIV) and tuberculosis (TB). HIV infection is a known risk factor for the development of active TB and it challenges in diagnosis and treatment of TB. Thus, it is essential to determine the epidemiology of HIV infection among TB patients to guide clinical actions and inform the policy makers. This study was aimed to assess the prevalence of HIV infection among TB patients and to describe the associated risk factors for HIV seropositivity.

**Methods:**

A retrospective study was conducted on TB registries at Metema Hospital directly observed therapy short-course (DOTS) clinic. Binary and multivariate logistic regression analysis was used to determine the association of HIV seropositivity among TB patients. Odds ratio (OR) and 95 % confidence intervals (CI) were calculated. P value less than 0.05 was considered as statistically significant.

**Results:**

Of the total 2096 pateints, 2005 (95.7 %) were tested for HIV. The overall HIV–TB co-infection rate was 20.1 % (404), 12.3 % (246) in males and 7.9 % (158) in females. The highest proportion of co-infection rate was observed among the patients in the age group of 25–34 years (32.4 %) and smear negative pulmonary TB patients (59.7 %). A declining trend of HIV–TB co-infection was observed during the study period, from 22.1 % (185) in 2009/10 to 12.8 % (52) in 2011/12 (X^2^ = 17.07, P < 0.001).

**Conclusions:**

This study found that HIV–TB co-infection is still high in the Metema area; and occurs more frequently in males than females, and among patients in age group of 25–34 years. Thus, concerted efforts and interventions methods that target these at risk groups are recommended.

## Background

Since the late 1980s following the human immunodeficiency virus (HIV) pandemic, tuberculosis (TB) has re-emerged as an important public health concern in the world. TB is the second largest killer of mankind of all infectious diseases following HIV/AIDS and it is the leading causes of death among HIV-infected patients [1]. The latest estimates of the World Health Organization (WHO) showed that TB is responsible for 9.0 million new cases and about 1.5 million deaths worldwide, of which, 360,000 deaths were among HIV positive people [2, 3]. A disproportionately large dual TB-HIV epidemic still exists in sub-Saharan Africa, accounting for about 80 % of the estimated global burden in 2013 [3].

HIV infection has been reported to associate with an increased risk of developing active TB by facilitating disease progression during primary TB infections or reactivation of latent infection [4–6]. Compared to HIV-uninfected people, for instance, HIV-infected people have been reported to be at about 35–40 times higher risk of developing active TB in newly acquired infection [7, 8]; and at about 20–30 times higher likelihood of reactivating latent infection [5, 6]. Further, HIV infection associates with atypical clinical presentation of TB including smear negative pulmonary TB, normal chest X-ray and higher frequency of extra-pulmonary TB (EPTB); thus, challenging TB diagnosis in middle and low-income countries [9]. Tuberculosis, on the other hand, associates with a chronic immune reactivation that increases the risk of HIV progression and death [10]. Thus, it is imperative to determine the prevalence of HIV infection among TB patients for proper clinical managements of both infections.

Ethiopia is one of the countries that are highly affected by dual epidemics of TB and HIV. In 2013, the prevalence and incidence rate of all forms of TB were 211 and 224 per 100,000 of the population, respectively [2]. The prevalence of TB–HIV co-infection among patients with known HIV status was 11 %. These data indicating that TB and HIV infections continue to be the major concerns of public health in Ethiopia, despite the country has achieved a considerable reduction of incidences and mortality resulting from these infections.

The epidemiology of HIV in Ethiopia shows remarkable variations with geographic regions, the study population and with the study periods. Although data on HIV prevalence and the associated risk factors among TB patients are documented from different parts of the country, information regarding this disease epidemiology from the Metema area is lacking [11–14]. Metema is located in Northwest Ethiopia on the Ethio-Sudan border. The major risk factor for HIV infection in Metema is related to the large numbers of seasonal migrant day laborers (over 80,000), which are the hardest hit by HIV, working in big commercial farms [15, 16]. The other important risk factor is that Metema is the center for people who cross the border for business and entertainment. This has attracted a large numbers of commercial sex workers (CSWs), who anticipate “good business” from local and foreign clients, into the town. Further, it had been speculated that HIV positive individuals from different parts of the country may migrate to the border towns like Metema to avoid social stigma surrounding the infection in their original residence places. Thus, there is a pressing need for continues evaluation of the magnitude of HIV infections among TB patients in the Metema area to generate pragmatic data for policy makers and to guide clinical actions.

To this end, the current study aims to assess the prevalence of HIV infection among TB patients who attended Metema Hospital, Northwest Ethiopia, and to describe associated risk factors for HIV infections.

## Methods

### Study settings and design

A retrospective study was conducted on TB registries at Metema hospital DOTS clinic to determine the prevalence of HIV infection and the associated risk factors. Metema district is located in North Gondar province of the Amhara regional state, 850 km from the country’s capital Addis Ababa. The town has a population of about 119,054 (63,433 male and 55,617 female) [18]. There are over 80,000 seasonal migrant workers (exclusively males) travelling to Metema for temporary labor; and return to their original residence places after 3–6 months of stay every year. The town is also noted for its border with Sudan, Ethio-Sudan highway, safe overnight truck stop, market and trading center, red light district and drinking houses, and bus station where porters, drivers, food and tea sellers, and most unemployed teen age boys and girls interact.

Metema hospital provides services for over 1500 patient in the Northwest Ethiopia per year. In the DOTS clinic, TB patients are treated and monitored as per the national tuberculosis and leprosy control program (NTLCP) guidelines [17]. There are also TB/HIV collaborative activities at the hospital which aim to reduce the burden of TB among people living with HIV (PLHIV) and to reduce the HIV/AIDS burden among TB patients. The activities of the collaborative program includes, (1) joint TB/HIV planning for integrated TB and HIV services delivery; (2) earlier initiation of ART and Isoniazid preventive therapy to prevent TB; (3) HIV testing and counselling to presumptive and confirmed TB patients, (4) HIV prevention interventions for presumptive and confirmed TB patients, and (5) provides cotrimoxazole preventive therapy for HIV positive TB patients.

### Study population and period

The study population was all TB patients who attended the Metema hospital between September 2009 and August 2012.

### Tuberculosis and HIV diagnosis

Tuberculosis diagnosis were performed by examining morning sputum samples with Zeihel–Nielsen staining, for the presence of acid fast bacilli (AFB); and/or based on chest radiography or physician’s judgement to treat patients with full course of anti-tuberculosis chemotherapy. Extra-pulmonary tuberculosis cases were determined based on pathological examination and/or clinical symptoms compatible with TB.

HIV infection was detected according to the national algorism for HIV testing. Briefly, sera prepared from venous blood/whole blood were screened using a HIV (1  +  2) antibody Colloidal Gold (KHB, Shanghai Kehua Bio-engineering Co Ltd, China), followed by HIV 1/2 STAT-PAK^®^ (Chembio Diagnostics, USA) if positive. Where the result of STAT-PAK^®^ is discordant with KHB, Unigold™ HIV test kit (Trinity Biotech, Ireland), was used to determine the result.

### Definitions

*Smear positive pulmonary TB case*: defined as a patient who tested AFB positive (AFB+) with one or more initial sputum smear examination by direct microscopy, or AFB+ with one sputum examination and radiographic abnormalities consistent with pulmonary TB case.

*Smear negative pulmonary TB:* when a patient does not meet the above criteria for smear positive pulmonary case but with at least two sputum smear examinations negative for AFB, clinical symptoms suggestive of TB and radiographic abnormality consistent with active pulmonary TB, or culture positive but sputum smear negative results.

*EPTB*: was defined as a patient with tuberculosis of organs other than lungs with diagnosis based on one culture-positive specimen, or histological or strong clinical evidence consistent with active extra-pulmonary disease, followed by a decision by a clinician to treat with a full course of anti-tuberculosis chemotherapy.

### Data collection

Data were collected from the DOTS registration book developed by the NTLCP of Ethiopia. Variables such as, age, sex, types of TB (whether pulmonary or extra-pulmonary), and co-morbidity with HIV/AIDS were collected.

### Statistical analysis

Data were entered, cleaned and analysed using SPSS version 20 statistical package (SPSS, Chicago, IL, USA). Data cleaning was performed to check for the consistency and completeness of the data set. Data were summarized using frequencies and proportions to describe the study population in relation to relevant variables. Bivariate and multivariate analysis was used to identify significant predictors. The degree of association between independent and dependent variables was assessed using odds ratio (OR) with 95 % confidence interval (CI). P value of less than 0.05 was considered as statistically significant.

### Ethics statement

Ethical approval was obtained from the University of Gondar ethical review board. Informed consent was not obtained as it was a retrospective study. Patients’ identifying information was kept confidentially.

## Results

### Characteristics of the study participants

A total of 2096 TB patients were registered at Metema Hospital DOTS clinic between September 2009 and August 2012. Of these, 1246 (59.4 %) were male and 850 (40.6 %) were female. The median age (IQR) of the patients was 28 years (17.00). Six hundred thirty-three (30.2 %) patients were in the age group of 25–34 year. Of the total 2096 TB patients, 1326 (63.3 %) were smear negative pulmonary tuberculosis cases, followed by 471 (22.5 %) smear positive pulmonary tuberculosis and 299 (14.3 %) extra-pulmonary tuberculosis cases. With respect to entry category, about 89.5 % (1876) were new TB cases, while 5.6 % (118) and 4.9 % (102) were retreatment and transfer TB cases, respectively (Table 1).

**Table 1:** Socio-demographic and clinical characteristics of TB patients treated at Metema Hospital DOTS center, Northwest Ethiopia, 2009–2012

Characteristics	Frequency (N)	Percentage (%)
Sex		
Male	1246	59.4
Female	850	40.6
Age		
0–14	254	12.1
15–24	537	25.6
25–34	633	30.2
35–44	394	18.8
45–54	151	7.2
55–64	74	3.5
>65	53	2.5
Type of entry		
New	1876	89.5
Relapse	115	5.5
Return after default	1	0.0
Failure	2	0.1
Transfer	102	4.9
TB forms		
Smear positive PTB	471	22.5
Smear negative PTB	1326	63.3
EPTB	299	14.3

*PTB* pulmonary tuberculosis, *EPTB* extra-pulmonary tuberculosis

### Prevalence of HIV/TB co-infection and associated risk factors

**Fig. 1:**
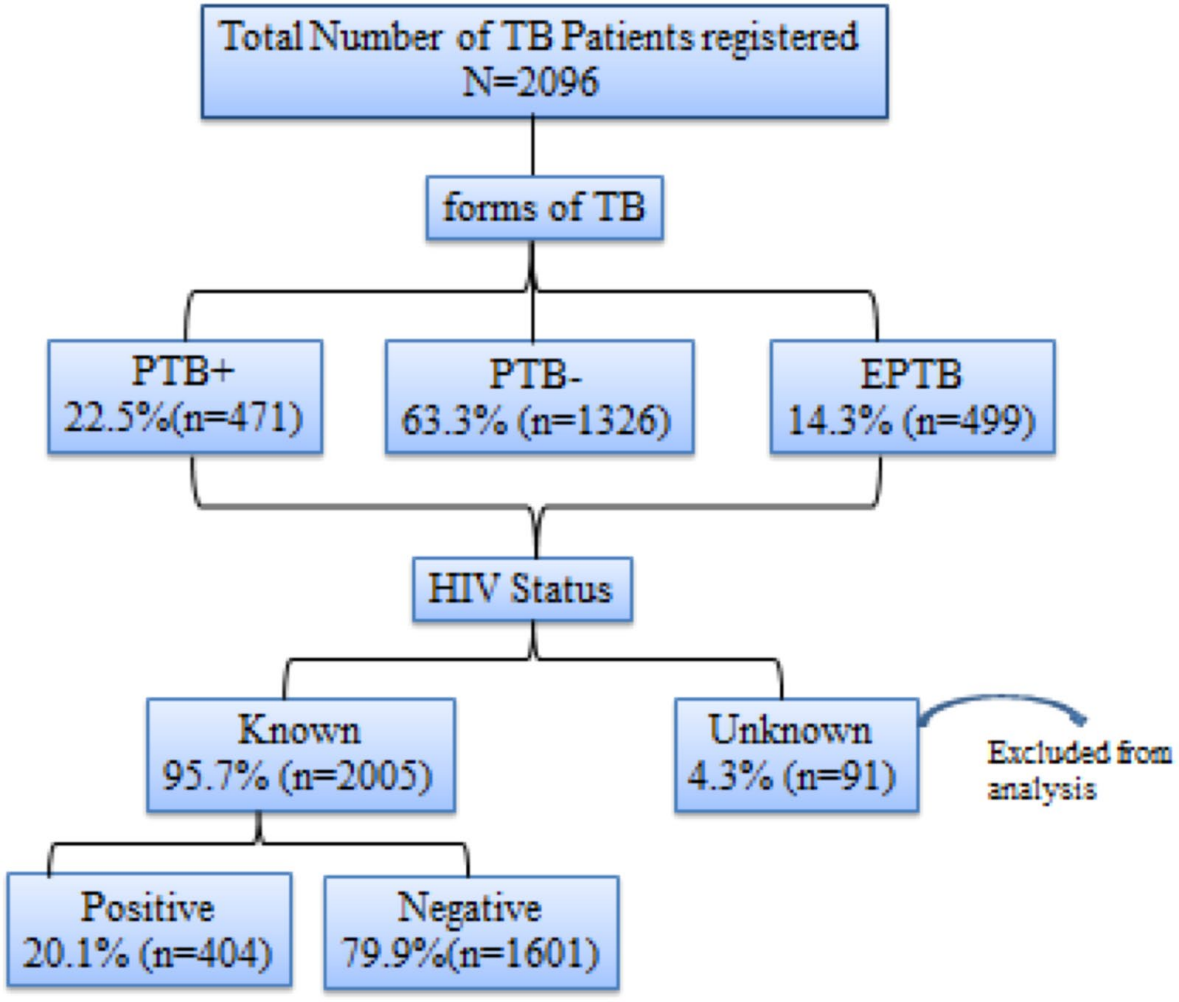
A flow chart showing characteristics of TB patients with respect to their HIV status and forms of tuberculosis. *TB* tuberculosis, *PTB+* smear positive pulmonary TB, *PTB−* smear negative pulmonary TB, *EPTB* extra-pulmonary TB

About 95.7 % (2005) of TB patients registered at DOTS center of Metema Hospital were tested for HIV, while 4.3 % (91) were not tested (Fig. 1). The trend of HIV–TB co-infection across the years is depicted in Table 2. A declining trend of HIV–TB co-infection was observed during the study period, from 22.1 % (185) in 2009/10 to 12.8 % (52) in 2011/12 (X^2^ = 17.07, P < 0.001). The overall prevalence of HIV–TB co-infection was 20.1 % (n = 404), 12.3 % (246) in males and 7.9 % (158) in females. In terms of age, the highest proportion of co-infection rate was observed among the patients in the age group of 25–34 years (32.4 %), followed by those in the age group of 15–24 years (26.5 %) and 35–44 years (17.1 %). With respect to TB forms, smear negative pulmonary TB patients constituted the higher proportion of HIV/TB co-infected patients (59.7 %) (Table 3).

**Table 2:** Trends of HIV–TB co-infection at Metema Hospital, September 2009–August 2012

Year	HIV status of TB Patients		Chi square	P value
	HIV–TB co-infection N (%)	TB only N (%)		
2009/2010	185 (22.1)	651 (77.9)	17.07	<0.001
2010/2011	167 (21.9)	596 (78.1)		
2011/2012	52 (12.8)	354 (68.2)		
Overall	404 (20.1)	1601 (79.9)

**Table 3:** TB patients at Metema Hospital DOTS center, according to HIV-infection status and associated risk factors for co-infection, Northwest Ethiopia, 2009–2012

Characteristics	HIV–TB N (%)	TB only N (%)	OR (95 % CI)	P value	AOR (95 % CI)	P value
Sex						
Male	246 (60.9)	940 (58.7)	1.09 (0.88–1.37)	0.43	1.1 (0.88–1.38)	0.41
Female	158 (39.1)	661 (41.3)	1.00	–	1.00	–
Age						
0–14	51 (12.6)	188 (11.7)	0.99 (0.68–1.42)	0.94	1.0 (0.69–1.45)	0.99
15–24	107 (26.5)	409 (25.5)	0.95 (0.71–1.27)	0.73	0.96 (0.72–1.28)	0.77
25–34	131 (32.4)	476 (29.7)	1.00	–	1.00	–
35–44	71 (17.6)	307 (19.2)	0.84 (0.61–1.16)	0.29	0.85 (0.61–1.18)	0.32
45–54	22 (5.4)	122 (7.6)	0.66 (0.40–1.07)	0.09	0.66 (0.40–1.08)	0.10
55–64	13 (3.2)	57 (3.6)	0.83 (0.44–1.56)	0.56	0.83 (0.44–1.57)	0.57
>65	9 (2.2)	42 (2.6)	0.78 (0.37–1.64)	0.51	0.77 (0.36–1.62)	0.49
Type of entry						
New	350 (86.6)	1448 (90.4)	1.00	–	1.00	–
Relapse	28 (6.9)	81 (5.1)	1.43 (0.92–2.23)	0.12	1.31 (0.82–2.07)	0.26
Defaulter	0 (0.0)	1 (0.1)	–	–	–	–
Failure	1 (0.2)	1 (0.1)	4.14 (0.26–66.31)	0.32	4.22 (0.26–68.64)	0.31
Transfer	25 (6.2)	70 (4.4)	1.48 (0.92–2.36)	0.11	1.41 (0.87–2.29)	0.17
TB forms						
PTB+	105 (26.0)	345 (21.5)	1.00	–	1.00	–
PTB−	241 (59.7)	1030 (64.3)	1.19 (0.83–1.70)	0.36	1.19 (0.82–1.72)	0.37
EPTB	58 (14.4)	226 (14.1)	0.91 (0.66–1.26)	0.57	0.97 (0.70–1.35)	0.85
Total	404 (20.1)	1601 (79.9)	–	–	–	–

*COR* crude odds ratio, *AOR* adjusted odds ratio, *CI* confidence interval, *PTB+* smear positive pulmonary TB, *PTB−* smear negative pulmonary TB, *EPTB* extra-pulmonary TB

Univariate and multivariable logistic regression analysis was performed to identify socio-demographic and clinical predictors for HIV/TB co-infection among the study participants (Table 3). The results showed that there was no significant association between HIV/TB co-infection, and the selected demographic and clinical determinants (P > 0.05).

## Discussions

The current study found a high rate of HIV infection among TB patients registered at Metema hospital DOTS center. The finding 20.1 % HIV/TB co-infection rate in this study was higher than those reported from India (18.86 %), Brazil (19 %), and the national co-infection rate (11.0 %) [2, 19, 20]. This data indicating that Metema area is among high HIV-prevalent settings, which are characterized by HIV prevalence of greater than/equal to 5 % among tuberculosis patients [21]. Yet, the prevalence rate observed in this study was lower when compared to similar studies from Gondar (67 %), Bahir Dar (25 %) and Debre Markos (44 %), Ethiopia [22–24]. The lower prevalence of HIV–TB co-infection in Metema could be related to a higher proportion of rural dweller in the study population due to a relatively small size of the town compared to the above mentioned cities.

In this study, HIV–TB co-infection rate was slightly higher in male patients than females. This finding is inconsistent with a number of studies that showed females to be more prone to HIV infection than their male counterparts [25–27]. This discrepancy between our finding and others could be plausibly explained by the fact that more male daily laborers than females migrate to the Metema area and may display risky sexual behavior. However, thorough and careful investigations are required to identify the contributing factors for such discrepancies. A high rate of HIV–TB co-infection was also noted in the age group of 25–34 years (32.4 %) and among smear negative pulmonary TB patients (59.7 %). These were in line with a number of previous studies that reported similar findings [28, 29].

Our study revealed that there was a declining trend of HIV prevalence among TB patients which is in agreement with the national HIV prevalence trend. This could be attributed to the expansion of health education on HIV as well as TB prevention and control in the area via deployment of health extension worker. However, given that we have analysed the trend of only 3 years, we recommend further investigations which incorporate HIV–TB co-infection data of several years before a firm conclusion is drawn.

This study has some limitations including lack of inclusions of important variables such as education level, economic status, marital status, and CD4 count which might affect their status with respect to these infections. Despite the limitations, the results of this study provided a useful data on the prevalence of HIV among TB patients at Metema hospital. This finding may have an implication for policy makers and TB program managers to address the health care need of these vulnerable populations in this part of Ethiopia.

## Conclusions

This study found that HIV–TB co-infection is still high in the Metema area; and occurs more frequently in males than females, and among patients in age group of 25–34 years. Thus, concerted efforts and interventions methods that target these at risk groups are recommended.

## Abbreviations

AIDS: acquired immunodeficiency syndrome
DOTS: directly observed therapy short-course
NTLCP: National Tuberculosis and Leprosy Control Program
IQR: interquartile range
